# Automated Detection and Classification of Oral Lesions Using Deep Learning to Detect Oral Potentially Malignant Disorders

**DOI:** 10.3390/cancers13112766

**Published:** 2021-06-02

**Authors:** Gizem Tanriver, Merva Soluk Tekkesin, Onur Ergen

**Affiliations:** 1 Graduate School of Sciences and Engineering, Koc University, Sariyer, Istanbul 34450, Turkey; gizem.tanriver@cantab.net; 2 Department of Tumour Pathology, Oncology Institute, Istanbul University, Fatih, Istanbul 34093, Turkey; 3 Department of Electronics and Communications Engineering, Istanbul Technical University, Maslak, Istanbul 34469, Turkey

**Keywords:** oral potentially malignant disorders, leukoplakia, oral cancer, screening, deep learning, convolutional neural network, semantic segmentation, instance segmentation, object detection, classification

## Abstract

**Simple Summary:**

Oral cancer is the most common type of head and neck cancer worldwide. The detection of oral potentially malignant disorders, which carry a risk of developing into cancer, often provides the best chances for curing the disease and is therefore crucial for improving morbidity and mortality outcomes from oral cancer. In this study, we explored the potential applications of computer vision and deep learning techniques in the oral cancer domain within the scope of photographic images and investigated the prospects of an automated system for identifying oral potentially malignant disorders with a two-stage pipeline. Our preliminary results demonstrate the feasibility of deep learning-based approaches for the automated detection and classification of oral lesions in real time. The proposed model offers great potential as a low-cost and non-invasive tool that can support screening processes and improve the detection of oral potentially malignant disorders.

**Abstract:**

Oral cancer is the most common type of head and neck cancer worldwide, leading to approximately 177,757 deaths every year. When identified at early stages, oral cancers can achieve survival rates of up to 75–90%. However, the majority of the cases are diagnosed at an advanced stage mainly due to the lack of public awareness about oral cancer signs and the delays in referrals to oral cancer specialists. As early detection and treatment remain to be the most effective measures in improving oral cancer outcomes, the development of vision-based adjunctive technologies that can detect oral potentially malignant disorders (OPMDs), which carry a risk of cancer development, present significant opportunities for the oral cancer screening process. In this study, we explored the potential applications of computer vision techniques in the oral cancer domain within the scope of photographic images and investigated the prospects of an automated system for detecting OPMD. Exploiting the advancements in deep learning, a two-stage model was proposed to detect oral lesions with a detector network and classify the detected region into three categories (benign, OPMD, carcinoma) with a second-stage classifier network. Our preliminary results demonstrate the feasibility of deep learning-based approaches for the automated detection and classification of oral lesions in real time. The proposed model offers great potential as a low-cost and non-invasive tool that can support screening processes and improve detection of OPMD.

## 1. Introduction

Oral cancer is the most common type of head and neck cancer worldwide, with an estimated 377,713 new cases and 177,757 deaths in 2020 [1]. Surgery is the usual primary treatment and generally yields high treatment success, with overall survival rates reaching 75–90% in the early stages [2,3]. However, over 60% of the cases are diagnosed at an advanced stage and progress with high morbidity and mortality [2,4]. Considering the alarming incidence and mortality rates, oral cancer screening has been an important part of many healthcare programs, as a measure to improve early detection of oral cancer [5]. Oral squamous cell carcinoma (OSCC), which makes up over 90% of oral cancer cases, is often preceded by oral potentially malignant disorders (OPMD), such as leukoplakia and erythroplakia [6]. The detection of OPMD, which has a risk of malignant transformation, is of the utmost importance for reducing morbidity and mortality from oral cancer and has been the main focus of the screening programs [6]. However, the implementation of these programs, based on visual examination, has been found to be problematic in a real-world setting as they rely on primary care healthcare professionals, who are often not adequately trained or experienced to recognize these lesions [6,7]. The substantial heterogeneity in the appearance of oral lesions makes their identification very challenging for healthcare professionals and is considered to be the leading cause of delays in patient referrals to oral cancer specialists [7]. Besides, early-stage OSCC lesions and OPMD are typically asymptomatic and may appear as small, harmless lesions, leading to late presentation of patients and ultimately leading to further diagnostic delay.

Advances in the fields of computer vision and deep learning offer powerful methods to develop adjunctive technologies that can perform an automated screening of the oral cavity and provide feedback to healthcare professionals during patient examinations as well as to individuals for self-examination. The literature on image-based automated diagnosis of oral cancer has largely focused on the use of special imaging technologies, such as optical coherence tomography [8,9], hyperspectral imaging [10], and autofluorescence imaging [11,12,13,14,15,16]. On the other hand, there have been a handful of studies performed with white-light photographic images [17,18,19,20,21], most of which focus on the identification of certain types of oral lesions.

The identification of OPMD is crucial for improving early detection of oral cancer and therefore has an important role in the development of oral cancer screening tools. In this study, our aim was to explore the potential applications of various computer vision techniques to the oral cancer domain in the scope of photographic images and investigate the prospects of a deep learning-based automated system for oral cancer screening.

## 2. Materials and Methods

### 2.1. Dataset

The study was conducted in collaboration with the Oncology Institute at Istanbul University and approved by the Ethics Committee of Istanbul University (Protocol number:1489-19). Photographic images of oral lesions with histopathological results were collected from the archive of the department of Tumour Pathology, which formed the initial source of our dataset. The rest of the images were collected from publicly available sources using search engines (http://images.google.com (accessed on 27 August 2020), https://yandex.com/images/ (accessed on 27 August 2020)). The dataset comprises a diverse set of lesions coming from a wide range of oral diseases and anatomical regions. Each lesion is classified as ‘benign’, ‘OPMD’, or ‘carcinoma’ based on the disease involved and its risk of progressing into oral cancer, as shown in Table 1. For the carcinoma class, only OSCC is included in the dataset. Besides the heterogeneity of oral diseases, the images in the dataset exhibit considerable variability in quality (i.e., lighting, zoom, angle, sharpness) and resolution.

The lesion instances were annotated by an expert oral pathologist (MST) using the VGG Image Annotator (VIA) tool [22,23]. Bounding polygons were drawn around the lesion areas and the corresponding class values were added as a region attribute. Our final dataset for the object detection experiments consisted of 652 images, which were split into approximately 80% for training, 10% for validation, and 10% for testing in a stratified fashion by maintaining the same proportion of class labels as in the original dataset. For the classification task, it was considered more practical to classify close-up lesion areas individually instead of assigning a global class for an entire image, since some images contain multiple lesions. Therefore, the lesion areas were rectangle-cropped, and these target regions were used for the classification experiments. This approach helps not only to boost the number of images for the classification experiments but also helps with the model training since oral lesions are often obscured by a complex background and structures such as teeth and dental tools. The distribution of images based on lesion class and dataset type is provided in Table 2.

The median width and height of the images in our dataset were 546 and 397 pixels, respectively. The images were resized to approximately 512 pixels along the longest image dimension for the segmentation and detection experiments. For the classification experiments, the cropped lesion areas were resized to respective input sizes based on the pretrained model, as explained in Section 2.5. Zero-padding was applied during resizing to preserve the aspect ratio of the image.

### 2.2. Semantic Segmentation Experiments

**Figure 1:**
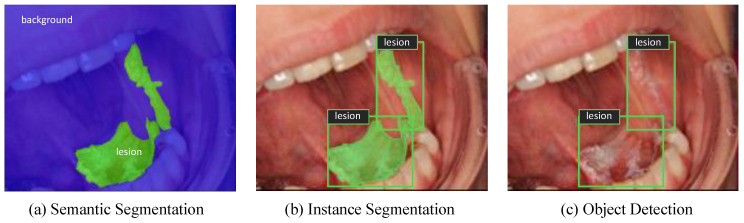
Different types of image recognition tasks showing (**a**) Semantic Segmentation, (**b**) Instance Segmentation, and (**c**) Object Detection.

Semantic segmentation is one of the image recognition tasks that deals with assigning each pixel of an image to a particular class including background, as shown in Figure 1a [24]. It is an important part of the automated diagnostic systems as it serves to delineate structural features such as boundaries of a lesion and provide a pixel-wise segmentation of anatomical structures. U-Net is a well-known segmentation model developed for biomedical image segmentation [25]. In order to segment oral lesion areas from their background, U-Net architecture was employed but with the encoder part replaced by an advanced convolutional backbone. Qubvel’s segmentation library, built on PyTorch framework (v1.7), was used for implementing the U-Net based models [26]. Data augmentation and transfer learning were utilized to overcome overfitting of the models. Test-time augmentation (TTA) was also implemented to improve predictions at test time. Further details of the experimental setup are available in Text S1. The performance of the models was measured based on the dice coefficient score (*F*_1_-measure) which is a frequently used metric for assessing pixel-level segmentation accuracy (Equation (1)). It is computed as the size of the overlap between the predicted mask and the ground-truth mask divided by the total size of the two masks.

(1) $$Dice\;Score=\frac{2\;TP\;}{2\;TP+FP+FN}\;$$

### 2.3. Instance Segmentation Experiments

While semantic segmentation is useful for delineating lesion areas from the images of the oral cavity, it does not differentiate between different lesion instances that may occur in an image. As shown in Figure 1b, instance segmentation provides an instance-level recognition by combining object detection and semantic segmentation tasks simultaneously. Mask R-CNN is one of the most well-known instance segmentation frameworks, which was developed by Facebook AI Research in 2017 [27]. It predicts a bounding box, a class label, and a pixel-level mask for each object instance separately. Facebook’s Detectron2 library was utilized for implementing Mask R-CNN with three different ResNet Feature Pyramid Network (FPN) backbones [28]. Further details of the experimental setup are available in Text S2.

Average precision (AP) was used as the evaluation metric for the instance segmentation and object detection experiments. A detection is considered a True Positive (TP) if the Intersection over Union (IoU) between the ground truth and the prediction is greater than a given IoU threshold and its predicted class is correct; otherwise, it is a False Positive (FP). If an object is not detected at all, then it is treated as a False Negative (FN). When precision is computed at each recall level based on the number of TP, FP, and FN, the area under the precision-recall curve gives the AP. We compute the AP over a range of IoU (Equation (2))thresholds from 0.5 to 0.95 with a step size of 0.05 and take the average of these values to produce the final AP for a given class as in the COCO evaluation (Equation (3)) [29]. We also report AP calculated at a fixed IoU threshold of 0.5, which is abbreviated as AP50.

(2) $$AP\left[class,\;IoU\right]=\int precision_{interp}\left(r\right)\;dr$$

(3) $$AP\left[class\right]=\frac{1\;}{Number\;of\;IoU\;thresholds}\underset{IoU}{\sum }AP\left[class,\;IoU\right]$$

### 2.4. Object Detection Experiments

Object detection is an area of computer vision that is concerned with detecting objects in images and videos, as illustrated in Figure 1c. YOLO architecture belongs to a family of single-stage object detectors which combines localization and classification tasks in a single network and therefore runs very fast thanks to its simple architecture [30]. YOLOv5 (PyTorch implementation of YOLOv4) was employed for lesion detection, which stands as the state-of-the-art algorithm for object detection in real time [31,32]. YOLOv5 is available in four versions based on the width and the depth of the network: YOLOv5s, YOLOv5m, YOLOv5l, and YOLOv5x. All of the four versions of the model were evaluated for the lesion detection task and were initialized with pretrained weights on the COCO dataset in order to prevent overfitting. Mosaic data augmentation was utilized by combining up to four images into one during training to improve detection of small-scale objects. Moreover, TTA and model ensembling (i.e., of multiple YOLOv5 versions) were implemented to improve performance at test time. Further details of the experimental setup are available in Text S3. The model performance is evaluated in terms of AP metric as in Mask R-CNN experiments.

### 2.5. Classification Experiments

Since oral lesions are often obstructed by structures such as teeth and dental tools, and some images contain multiple lesions of different classes, lesion areas were isolated as explained in Section 2.1 and classification experiments were performed on these cropped images. Various Convolutional Neural Network (CNN) architectures were evaluated for the lesion classification task, such as ResNet-152 [33], DenseNet-161 [34], Inception-v4 [35], and EfficientNet-b4 [36]. Additionally, an ensemble model of DenseNet161 and ResNet-152 was built, which uses the average of the outputs produced by two models for prediction. Images were resized to 224 pixels for ResNet-152, DenseNet-161, and the average ensemble models, 299 pixels for Inception-v4, and 380 pixels for EfficientNet-b4 model. All models were initialized with weights pretrained on the ImageNet dataset provided by open-source PyTorch libraries [37,38,39]. The same data augmentation techniques were utilized across the models. Further details of the experimental setup are available in Text S4 and Table S2.

We computed the confusion matrices on the test set to summarize model predictions. Performance metrics such as precision, recall, and *F*_1_ score were computed as described in Equation (4), Equation (5), and Equation (6). To account for class imbalance, we reported a weighted macro-average *F*_1_-score, which is the weighted average of *F*_1_-scores for each class. Confusion matrices and related metrics were calculated using scikit-learn library [40].

(4) $$\begin{array}{c} Precision=\frac{TP\;}{TP+FP} \end{array}$$

(5) $$\begin{array}{c} Recall=\frac{TP\;}{TP+FN} \end{array}$$

(6) $$\begin{array}{c} F_{1}=\frac{2\times Precision\times Recall\;}{Precision+Recall} \end{array}$$

## 3. Results

### 3.1. Semantic Segmentation Experiments

**Figure 2:**
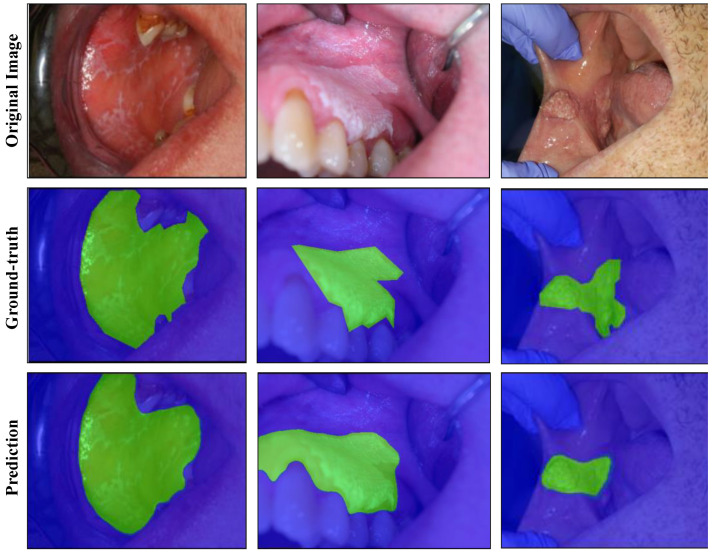
Semantic segmentation of oral lesions with EfficientNet-b7 model on test set. Each column is displaying a single test example with the original image in the top row, the ground-truth segmentation in the middle row, and the predicted segmentation in the bottom row. Lesion and background masks are shown in green and blue colours, respectively.

U-Net architecture was evaluated with different convolutional backbones for the lesion segmentation task. Hyperparameters were optimized based on the performance on the validation set (details available in Table S1 and Figure S1). The results for the background vs. lesion segmentation task are shown in Table 3. The EfficientNet-b7 model achieved a dice score of 0.926 without TTA and 0.929 with TTA. The predicted mask outputs and their corresponding ground-truth masks are provided for the test images in Figure 2, using the best performing model.

### 3.2. Instance Segmentation Experiments

**Figure 3:**
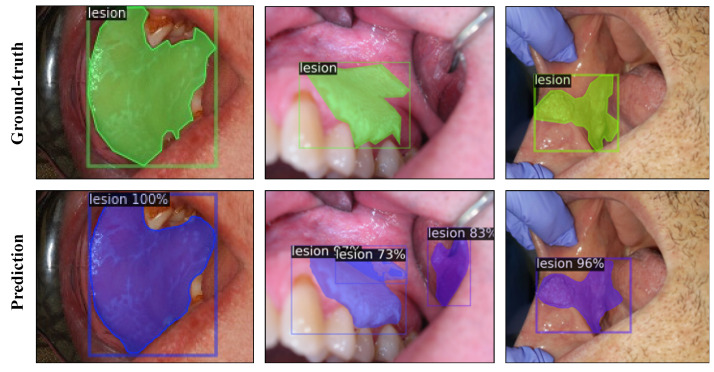
Instance segmentation of oral lesions with Mask R-CNN ResNeXt-FPN model showing ground-truth (top row) and predicted (bottom row) bounding box and segmentation masks for test images. The values printed on top of the predicted boxes are the confidence scores for each prediction.

The test results of Mask R-CNN experiments with ResNet-50, ResNet-101, and ResNeXt-101 FPN backbones are presented in Table 4 with and without TTA. The learning curve plots are provided in Figure S2. The ResNeXt-101 FPN model achieved an AP score of 43.90 for box detection and 37.85 for mask detection without TTA on the test set. At the fixed IoU threshold of 0.5, the ResNeXt-101 FPN model achieved AP scores of 79.74 and 78.00 for box and mask detection, respectively. The model predictions are illustrated for the test images in Figure 3, using the best performing model.

### 3.3. Object Detection Experiments

**Figure 4:**
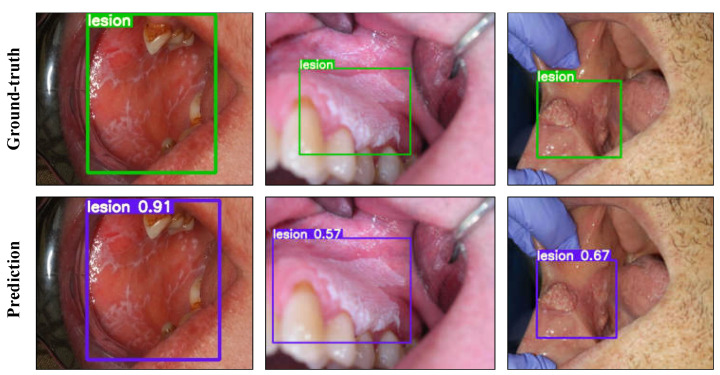
Lesion detection results with YOLOv5l on the test set. The top and bottom rows show the ground-truth boxes and the model predictions, respectively. The values printed on top of the predicted boxes are the confidence scores for each predicted box.

Different versions of YOLOv5 were evaluated for the one-class lesion detection task. Model hyperparameters were optimized based on the performance on the validation set. The learning curve plots are provided in Figure S3 and the test results of each model are reported in Table 5. The model outputs are presented in Figure 4 using YOLOv5l with selected confidence and non-max suppression (NMS) thresholds.

### 3.4. Classification Experiments

**Figure 5:**
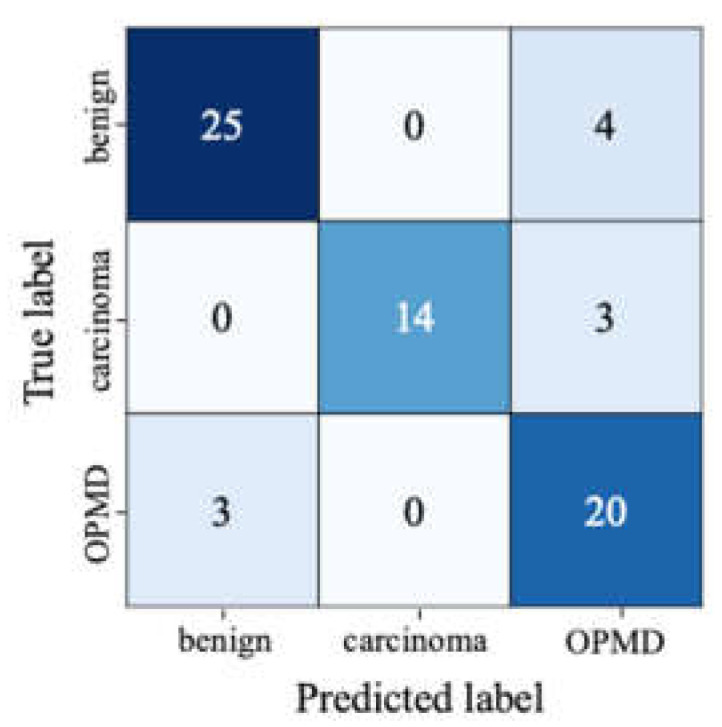
Confusion matrix for the EfficientNet-b4 model on the test set. The values on the diagonal axis show the number of TP and TN predictions and the off-diagonal values show the number of FP and FN predictions, respectively, for each class.

**Figure 6:**
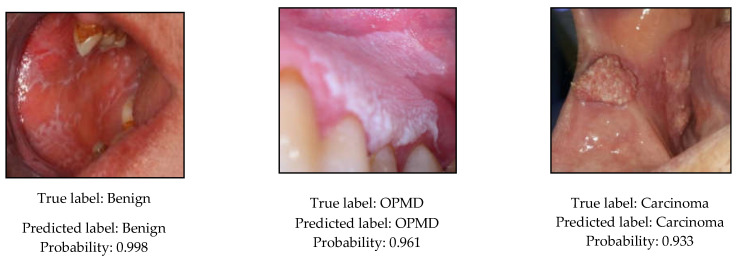
Predicted class labels and probabilities for the test images by the EfficientNet-b4 classification model.

The precision, recall, and *F*_1_-scores of the evaluated CNN models are reported in Table 6 on the test set. Furthermore, confusion matrix and per-class precision and recall results are provided for the EfficientNet-b4 model in Figure 5 and Table 7. Moreover, the predicted class probabilities and the true labels of the test images are displayed in Figure 6 or the EfficientNet-b4 model.

## 4. Discussion

Oral cancer is a disease that has recently increased worldwide but is still not fully understood. OSCC accounts for more than 90% of oral cancers. The cause of OSCC is multifactorial, including extrinsic factors such as tobacco products and alcohol and intrinsic factors such as malnutrition and iron-deficiency anaemia [41]. Many OSCCs have been documented to be associated with or preceded by OPMD, especially leukoplakia [41,42]. Therefore, correct classification of oral lesions, especially white ones, is critical in clinical practice.

Exploiting the advancements in deep learning, a two-stage model is presented in this study to detect oral lesions with a detector network and classify the detected region as benign, OPMD, or carcinoma, based on the risk of malignant transformation with a second-stage classifier network. The proposed model can enable the detection of oral lesions, including benign and OPMD, in real time, and presents significant opportunities for the development of a vision-based oral cancer screening tool.

Segmentation and/or detection of lesion areas is an essential step for the identification of oral lesions. Therefore, several segmentation and detection algorithms were evaluated to isolate the region of interest, i.e., the lesion area, from photographic images of oral lesions. For pixel-wise semantic segmentation, U-Net models performed well on the segmentation task with the EfficientNet-b7 model achieving the highest dice score of 0.929. As shown in Figure 2, lesions of various types and sizes were segmented with good precision. To our knowledge, this is the first report of semantic segmentation of oral lesions using photographic images. While the results with U-Net-based models are very promising, the application of semantic segmentation may be limited in certain tasks, as it treats multiple lesion instances in an image as a single lesion object. In that respect, instance segmentation may be more applicable, which aims to detect and segment each lesion instance separately.

The capability of the well-known Mask R-CNN architecture was studied for the instance segmentation of oral lesions from photographic images. In experiments with Mask R-CNN, using different ResNet backbones, all models achieved similar results in terms of box and mask AP on the instance segmentation task. Overall, the best performance without TTA was obtained by the ResNeXt-101 model in line with the reported results on the COCO dataset [28]. Despite a few false positive and negative predictions, the model demonstrated the capability of detecting and segmenting lesions simultaneously with good precision. The model also performed well with challenging cases such as when the lesion was occluded in the oral cavity or had a relatively smaller size with respect to the image size. One drawback of Mask R-CNN is slow inference speed due to its complex architecture. While it can be useful for applications that do not require real-time inference, it falls behind many object-detection models in terms of inference speed.

As a single-stage object detector, the performance of the state-of-the-art YOLOv5 architecture was evaluated for the detection of oral lesions. The YOLOv5l model performed the best among all versions, with an AP of 0.644 and an AP50 of 0.951 on the test set and an inference speed of 10.6 ms per image on Tesla T4 graphics processing unit (GPU). With TTA applied, YOLOv5l achieved 0.953 on AP50 although this comes at a cost of slight reduction in AP since TTA generally works to increase recall at a cost of reduced precision. As expected, the model performance improved with bigger models, with the exception of YOLOv5x, which was prone to overfitting during training and achieved a lower AP compared to YOLOv5l on the test set. This could be due to the small size of our dataset, which is better suited to less complex models on this task. We further showed that the ensemble of two smaller models such as YOLOv5s and YOLOv5m can achieve almost the same AP as YOLOv5l but at a higher inference speed. With a very high inference speed and low computational cost, YOLOv5 offers great potential for deployment in a real-world application.

Different types of CNN models, including an ensemble model of DenseNet-161 and ResNet-152, were evaluated for the multi-class classification of oral lesions based on the risk of malignant transformation. Among all models, EfficientNet-b4 and Inception-v4 achieved the highest *F_1_*-score of 0.855 on the test set. The larger input dimensions and the advanced architectures of these models were likely to have provided performance gains over the other models that were evaluated for the classification task. We further analysed the results of EfficientNet-b4, which outperformed other models in terms of model complexity, computational cost, and accuracy combined. As shown in Table 7, the OPMD class had the lowest precision, with four benign and three carcinoma lesions misclassified as OPMD. Misclassification of carcinoma lesions as OPMD may not pose a significant risk since both types of lesions should be referred to a medical professional immediately. On the other hand, misclassification of benign lesions as OPMD may lead to increased referrals to oral cancer specialists and present an additional burden on the clinical staff. Nevertheless, the recall for OPMD was relatively high, which is encouraging since the detection of OPMD is of great importance for oral cancer screening.

The scope of the studies in the related literature have mostly been limited to certain types of oral lesions, such as mouth sores [17,18] or tongue lesions [19], which represent only a small fraction of the oral lesions. In a more recent study by Welikala et al., the authors investigated the feasibility of deep learning methods for detection and classification of oral lesions based on referral decisions using a more comprehensive dataset [20]. Although the results were encouraging, particularly for the binary classification of oral lesions as “non-referral” or “referral”, the models did not achieve good performance on the detection and multi-class classification of oral lesions. The classifiers were trained with the whole images of the oral cavity, instead of the target lesion areas, which may have impacted the accuracy of the models. Recently, Fu et al. proposed to use cascaded deep networks to detect OSCC from photographic images and compared the performance of the model with that of human readers [21]. The authors used a pretrained detector network as a pre-processing step to crop the lesion area and a classifier network to classify the candidate patches as oral cancer or normal mucosa. While the proposed model was shown to perform on par with human experts in identifying OSCC lesions, the study failed to investigate the model’s capacity for differentiating between OSCC and non-OSCC oral diseases or between non-OSCC oral diseases and normal oral mucosa. In this work, we demonstrated the feasibility of classifying oral lesions into three categories based on their risk of progression into oral cancer, with reasonable precision and recall across all classes despite the size of our dataset and the variability of images. Unlike previous work, we utilized more recent and advanced architectures for classification, such as Inception-v4 and EfficientNet-b4, which provided significant improvements in evaluated metrics. As the models are trained with more images representing all the clinical variations of oral lesions, the model performance is expected to further improve significantly.

**Figure 7:**
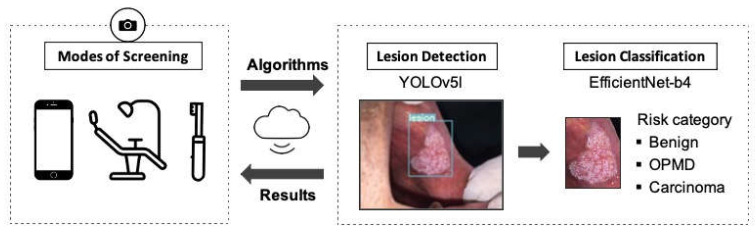
Proposed two-stage pipeline for oral cancer screening. It includes the detection of oral lesions from photographic images in the first stage and the classification of the detection region in the second stage using deep learning algorithms. YOLOv5l and EfficientNet-b4 models were selected for the pipeline as they achieved overall good performance in terms of both accuracy and inference time.

Finally, we proposed an end-to-end, two-stage model for identifying oral lesions by combining object detection and classification tasks in a serial manner. In our proposed pipeline, YOLOv5l is used for detecting lesion regions from the whole image and EfficientNet-b4 is used for classifying the detected lesion region into three categories. The selected networks achieved overall good performance in terms of both accuracy and inference time, which makes them highly suitable for deployment in a real-time application. As illustrated in Figure 7, the proposed model offers great potential for automated identification of various oral lesion types and paves the way for a low-cost, non-invasive, and easy-to-use oral cancer screening tool. It can be deployed as a mobile application for self-screening of the oral cavity or to support the oral cancer screening process at primary care centres, such as dental practices. Moreover, it could be used for remote follow-up of patients who have undergone surgery or received treatment for any disease recurrence.

## 5. Conclusions

This study presents the potential applications of deep learning algorithms for segmentation, detection, and multi-class classification of oral lesions as benign, OPMD, or carcinoma. Based on the results obtained, a two-stage deep learning pipeline is proposed for oral cancer screening which enables automated detection and classification of various oral lesion types in real time with low computational costs. We envisage that the proposed model paves the way for a low-cost, non-invasive, and easy-to-use tool that can support screening processes and improve detection of OPMD. A larger dataset with more examples of challenging lesion types can bring significant gains to all of the models; therefore, it will be the focus of future studies.

## Figures and Tables

**Table 1:** Lesion classes and corresponding oral diseases included within each class.

Class	Disease	Number of Lesions
Benign	Dermatologic diseases (geographic tongue, lichen planus, systemic lupus erythematosus, pemphigoid, erythema multiforme, pemphigus vulgaris)	90
	Fungal diseases (median rhomboid glossitis, candidal leukoplakia, pseudomembranous candidiasis)	33
	Inflammatory process (nicotine stomatitis, gingivitis, periodontitis, pericoronitis)	30
	Developmental defects (fissured tongue, thrush, hairy tongue, leukoedema, Fordyce granules)	24
	Ulcers (aphthous ulcer, traumatic ulcer, viral ulcers, TUGSE)	54
	Keratosis (reactive / traumatic keratosis, linea alba)	36
	Hairy leukoplakia	7
OPMD	Leukoplakia	156
	Erythroplakia	35
	Erythroleukoplakia	46
	Submucous fibrosis	11
Carcinoma	Squamous cell carcinoma	162

**Table 2:** Number of images for classification experiments according to lesion class and dataset type.

Dataset Type	Benign	OPMD	Carcinoma	Total Number of Lesions
Training	219	203	130	552
Validation	26	22	15	63
Test	29	23	17	69
-	274	248	162	684

**Table 3:** Test results of U-Net with various backbones for the background vs. lesion segmentation task. The models were evaluated based on the dice (F_1_) score as the evaluation metric using the best model checkpoints.

Backbone	Dice_test_	Dice_test_ with TTA
EfficientNet-b3	0.925	0.927
Densenet-161	0.921	0.927
Inception-v4	0.915	0.922
EfficientNet-b7	0.926	0.929
ResNeXt-101_32x8d	0.923	0.928

**Table 4:** Mask-RCNN results on test set with ResNet-50, ResNet-101, and ResNeXt-101 FPN backbones. The results are provided with and without TTA. Speed_GPU_ measures per image inference speed in milliseconds (ms) using one Tesla T4 GPU and includes image pre-processing, inferencing, post-processing and NMS.

Backbone	Box AP	Box AP50	Mask AP	Mask AP50	Speed_GPU_
ResNet-50 FPN	42.53	80.51	37.23	74.08	46
ResNet-50 FPN + TTA	42.65	82.63	37.98	76.19	361
ResNet-101 FPN	41.85	81.86	37.70	74.41	56
ResNet-101 FPN + TTA	40.54	83.64	37.52	72.96	442
ResNeXt-101 FPN	43.90	79.74	37.85	78.00	89
ResNeXt-101 FPN + TTA	43.35	81.60	37.80	78.92	786

**Table 5:** YOLOv5 results for lesion detection with the best model checkpoints on the test set. Speed_GPU_ measures per image inference speed in milliseconds (ms) using one Tesla T4 graphics processing unit (GPU) and includes image pre-processing, inferencing, post-processing, and non-max suppression.

Model	AP	AP50	Speed_GPU_
YOLOv5s	0.579	0.920	4.4
YOLOv5m	0.607	0.896	6.9
YOLOv5l	0.644	0.951	10.6
YOLOv5l + TTA	0.622	0.953	21.2
YOLOv5x	0.613	0.902	18
YOLOv5x + TTA	0.630	0.940	35.3
YOLOv5s & 5m ensemble	0.637	0.923	9

**Table 6:** Classification results of different CNN models on the test set of cropped lesion regions with the best model checkpoints. Precision, recall, and *F*_1_-score are reported as weighted macro-averages.

Model	Input Size	Precision	Recall	F_1_-Score
EfficientNet-b4	380	0.869	0.855	0.858
Inception-v4	299	0.877	0.855	0.858
DenseNet-161	224	0.879	0.841	0.844
Ensemble	224	0.849	0.841	0.843
ResNet-152	224	0.826	0.812	0.811

**Table 7:** Class-wise precision, recall, and F1-score results for the test set with the EfficientNet-b4 model.

Class	Precision	Recall	F_1_-score	Support
Benign	0.89	0.86	0.88	29
OPMD	0.74	0.87	0.90	23
Carcinoma	1.00	0.82	0.90	17
Weighted average	0.87	0.86	0.86	69

## Data Availability

The data are available from the corresponding authors upon reasonable request within IRB rules.

## Supplementary Materials

The following are available online at https://www.mdpi.com/article/10.3390/cancers13112766/s1, Text S1: Semantic Segmentation: U-Net Experiments, Table S1: Backbones implemented with U-Net, number of model parameters (in million) and selected hyperparameters such as batch size and learning rate are shown for each model, Figure S1: Training plots showing dice loss and dice score on the validation set during training of the U-Net models with different backbones, Text S2: Instance Segmentation: Mask R-CNN Experiments, Figure S2: The learning curves for the Mask R-CNN models showing box and mask AP results on the validation set over 10,000 iterations, Text S3: Object Detection: YOLOv5 Experiments, Figure S3: The learning curves for the YOLOv5 models showing AP (left) and AP50 (right) on the validation set over 80 epochs, Text S4: Classification Experiments, Table S2: Selected hyperparameters for the classification experiments.
