# Supplementary material for: Automated Detection and Classification of Oral Lesions Using Deep Learning to Detect Oral Potentially Malignant Disorders
Source: Cancers (Basel). 2021 Jun 2;13(11):2766. doi: 10.3390/cancers13112766 (PMC8199603; doi:10.3390/cancers13112766)
Supplement: Supplementary file 1 [file cancers-13-02766-s001.zip › cancers-1171303-supplementary.pdf]

cancers

MDPI

Supplementary Material: Automated Detection and Classification of Oral Lesions Using Deep Learning to Detect Oral Potentially Malignant Disorders

Gizem Tanriver, Merva Soluk Tekkesin and Onur Ergen

Text S1: Semantic Segmentation: U-Net Experiments

The dataset was prepared for the semantic segmentation experiments by generating masks for each image from polygon annotations, where each mask was a stack of Boolean arrays, one for each class. For all models, the images and their corresponding masks were resized to 512 × 512 pixels with zero-padding to retain the original aspect ratio. Data augmentation was applied to the training set, including horizontal and vertical flipping, random rotation, shifting, zooming, adding noise, sharpening, embossing, blurring, and colour space augmentations (i.e., brightness, saturation, and contrast) using albumentations library [1]. Qubvel's segmentation library was utilized for implementing U-Net with different backbones [2]. Due to our small dataset, we initialized all backbones with pretrained ImageNet weights. Images were normalized according to the data statistics of corresponding pretrained models. For training of the models, dice loss was used with an Adam optimizer. Hyperparameters were optimized on the validation set for each model and selected as shown in Supplementary Table 1. Following hyperparameter optimization, the final performance was evaluated on the test set. To further boost the test accuracy, test-time augmentation (TTA) was applied by testing the model on the augmented versions of the image (horizontal and vertical flips and rotations of 0, 90, 180, 270 angles) and taking the average of the results. All experiments were run on Tesla V100 graphics processing unit (GPU) with 32GB memory. The training plots are shown in Supplementary Figure 1.

Table S1. Backbones implemented with U-Net, number of model parameters (in million) and selected hyperparameters such as batch size and learning rate are shown for each model.

Encoder	Parameters	Batch Size	Learning Rate	Epochs
EfficientNet-b3	10M	8	0.0002	40
Densenet-161	26M	4	0.0001	40
Inception-v4	41M	8	0.0003	40
EfficientNet-b7	63M	4	0.0001	40
ResNeXt-101_32x8d	86M	12	0.0001	40

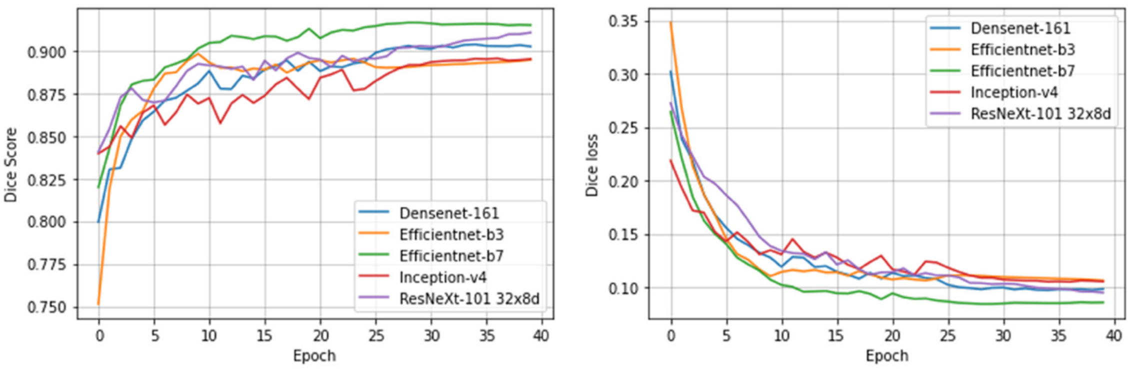

Figure S1. Training plots showing dice loss and dice score on the validation set during training of the U-Net models with different backbones. There is an exponential increase in dice score (and a decrease in dice loss) in the early phases of training, followed by saturation as the models converge to an optimal solution.

Text S2: Instance Segmentation: Mask R-CNN Experiments

Detectron2 library was used for implementing Mask R-CNN models [3]. All images were resized to 400 pixels along the shorter image dimension with a limit of 512 on the longer dimension. With regards to data augmentation, vertical and horizontal flipping, random cropping, random rotation at multiples of 90 degrees, and colour space augmentations were applied to the training data. Hyperparameters were optimized based on performance on the validation set. Adjustments to the anchor scales and aspect ratios were made according to the distribution of our data. As the relative area of the lesion instances in our dataset covers a wide range, from a minimum of approximately 38 pixels (i.e., sqrt of area) up to 512 pixels when resized, anchor scales were chosen as [[48, 64, 128, 256, 512]]. Similarly, the aspect ratios (i.e., height to width) of [[0.25, 0.5, 1.0, 2.0, 5.5]] were used in our implementation to account for the variation in our dataset, instead of the original aspect ratios of [[0.5, 1.0, 2.0]]. For the head of Mask R-CNN, a reduced ROI batch size of 256 was used to balance the number of foreground and background samples at a ratio of 1:3. Stochastic gradient descent (SGD) optimizer was selected with a learning rate of 0.0002, momentum of 0.9, and weight decay of 0.0005 based on optimization experiments. All models were run for 10,000 iterations (~76 epochs) at a batch size of 4 including initial warm up for ~3 epochs. Learning rate was decayed by 0.1 at iteration 6,000 for all models, with an additional earlier decay at iteration 3,000 for the ResNeXt-101 model. To prevent overfitting, all models were initialized with pretrained COCO weights and best model checkpointing was utilized. The freezing of ResNet stages (pretrained) was found to provide worse results on our dataset, therefore the entire backbones were fine-tuned. The training plots for each model are shown in Supplementary Figure 2. The non-max suppression (NMS) threshold and confidence threshold, which are used at test time to produce final outputs, were selected as 0.5 to eliminate highly overlapping and low score predictions. After hyperparameter optimization, the final performance of the models was evaluated on the test set. Results are reported with and without TTA which applies horizontal flipping and processing at 3 different resolutions (400, 500, 600 pixels) and takes the average of the augmented results.

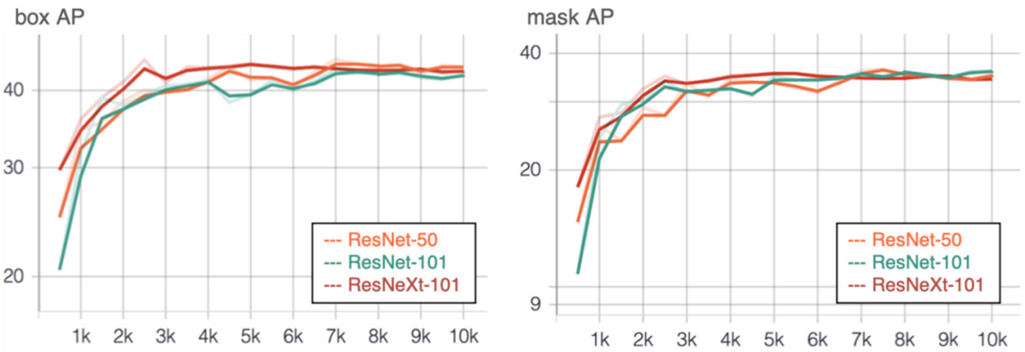

Figure S2. The learning curves for the Mask R-CNN models showing box and mask AP results on the validation set over 10,000 iterations. Metrics were computed at every 500 iterations due to computational overhead.

Text S3: Object Detection: YOLOv5 Experiments

The object detection models were developed using YOLOv5 implementation of ultralytics [4]. Images were resized to 512 pixels along the longest image dimension and padded along the shorter dimension to a minimum multiple of 32 for rectangular inference. During training, mosaic data augmentation as well as vertical and horizontal flipping, scaling, shifting, and colour space augmentations were applied to the training set. We used the Nesterov SGD optimizer with a learning rate of 0.001, momentum of 0.98, weight decay of 0.0005, and batch size of 8. Models were run for 80 epochs, including an initial warm up period of 3 epochs. At test time, we used a confidence threshold of 0.55

and an NMS threshold of 0.3 to produce final outputs. Furthermore, we utilized TTA by applying horizontal flipping and processing at 3 different resolutions (original resolution and 2 reduced multiples) on the test set, to improve recall of the models. Ensemble of multiple models, such as YOLOv5s and YOLOv5m, was also evaluated to further boost performance. Hyperparameters were selected based on performance on the validation set by using both random search and a genetic algorithm provided by the repository. We used an Nvidia Tesla V100 GPU with 32GB memory for training the models. The training plots are shown in Figure S3.

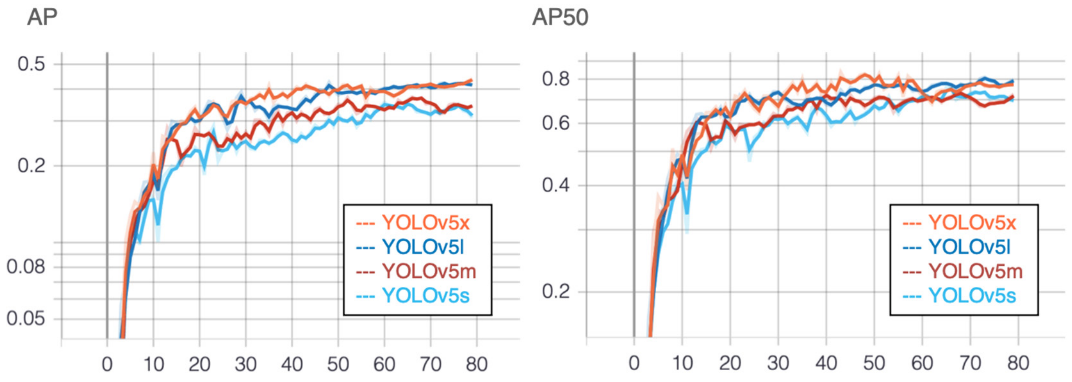

Figure S3. The learning curves for the YOLOv5 models showing AP (left) and AP50 (right) on the validation set over 80 epochs.

Text S4: Classification Experiments

Data augmentation such as horizontal and vertical flipping, rotation at multiples of 90 degrees, perspective distortion, shifting, shearing, blurring, adding noise, and augmenting colour space (i.e., brightness, saturation, contrast, and jitter) were applied at random to the training set using PyTorch's transform functions [5–7]. Images were normalized according to mean and standard deviation of the pretraining settings. Hyperparameters, including data augmentation hyperparameters, were optimized based on performance on the validation set and selected as shown in Supplementary Table 2. For DenseNet161 and ResNet-152 training, the models were fine-tuned in three stages – heads only, some layers (denseblock3 onwards in DenseNet161 and block 3.14 onwards ResNet-152), all layers – with learning rate reduction at every stage. Momentum was set as 0.9 for all experiments. Learning rate was reduced by a factor of 0.5 when validation loss plateaued for at least 3 epochs and the best model was saved during training in order to prevent overfitting. After hyperparameter optimization, the final performance was evaluated on the test set. All experiments were run with Torch 1.7 and Torchvision 0.8.1 with CUDA 10.1 using Nvidia Tesla T4 GPU with 16 GB memory.

Table S2. Selected hyperparameters for the classification experiments.

Model	Optimizer	Batch size	Learning rate	Epochs	Weight decay
**EfficientNet-b4**	Rmsprop	16	5e-6	35	1e-2
**Inception-v4**	SGD	16	1e-3	35	1e-3
**Ensemble**	SGD	16	1e-3	35	5e-4
**ResNet-152**	SGD	16	1e-3, 5e-4, 5e-5	3-12-20	5e-4
**DenseNet-161**	SGD	16	1e-3, 5e-4, 5e-5	3-12-20	1e-3

References

1. Buslaev, A.; Iglovikov, V. I.; Khvedchenya, E.; Parinov, A.; Druzhinin, M.; Kalinin, A.A. Albumentations: Fast and Flexible Image Augmentations. *Information* **2020**, *11*, 125, doi:10.3390/info11020125.
2. Yakubovskiy, P. Segmentation Models Pytorch. Available online: https://github.com/qubvel/segmentation_models.pytorch (accessed on 16 December 2020).

3. Wu, Y.; Kirillov, A.; Massa, F.; Lo, W.-Y.; Girshick, R. Detectron2. Available online: https://github.com/facebookresearch/detectron2 (accessed on 16 December 2020).
4. Jocher, G.; Stoken, A.; Borovec, J.; Christopher, S; Changyu, L.; Hogan, A.; Diaconu, L.; Poznanski, J.; Rai, P.; Ferriday, R.; et al. Ultralytics/Yolov5: V2.0. *GitHub* **2020**, doi:10.5281/ZENODO.3958273.
5. Paszke, A.; Gross, S.; Massa, F.; Lerer, A.; Bradbury, J.; Chanan, G.; Killeen, T.; Lin, Z.; Gimelshein, N.; Antiga, L.; Desmaison, A.; Köpf, A.; Yang, E.; DeVito, Z.; Raison, M.; Tejani, A.; Chilamkurthy, S.; Steiner, B.; Fang, L.; Bai, J.; Chintala, S. PyTorch: An Imperative Style, High-Performance Deep Learning Library. *Adv. Neural Inf. Process. Syst.* **2019**, 8024–8035.
6. Melas-Kyriazi, L. lukemelas/EfficientNet-PyTorch: A PyTorch implementation of EfficientNet. Available online: https://github.com/lukemelas/EfficientNet-PyTorch (accessed 3 December 2020).
7. Cadene, R. cadene/pretrained-models.pytorch: Pretrained ConvNets for pytorch. Available online: https://github.com/Cadene/pretrained-models.pytorch#modelinput_size (accessed 1 December 2020).

Publisher's Note: MDPI stays neutral with regard to jurisdictional claims in published maps and institutional affiliations.

© 2021 by the authors. Licensee MDPI, Basel, Switzerland. This article is an open access article distributed under the terms and conditions of the Creative Commons Attribution (CC BY) license (http://creativecommons.org/licenses/by/4.0/).
